# Assessment of a Digital Symptom Checker Tool's Accuracy in Suggesting Reproductive Health Conditions: Clinical Vignettes Study

**DOI:** 10.2196/46718

**Published:** 2023-12-05

**Authors:** Kimberly Peven, Aidan P Wickham, Octavia Wilks, Yusuf C Kaplan, Andrei Marhol, Saddif Ahmed, Ryan Bamford, Adam C Cunningham, Carley Prentice, András Meczner, Matthew Fenech, Stephen Gilbert, Anna Klepchukova, Sonia Ponzo, Liudmila Zhaunova

**Affiliations:** 1 Flo Health UK Limited London United Kingdom; 2 Your.MD Limited London United Kingdom; 3 Una Health GmbH Hamburg Germany; 4 Else Kröner Fresenius Center for Digital Health TUD Dresden University of Technology Dresden Germany

**Keywords:** women's health, symptom checkers, symptom checker, digital health, chatbot, accuracy, eHealth apps, mobile phone, mobile health, mHealth, mobile health app, polycystic ovary syndrome, gynecology, digital health tool, endometriosis, uterus, uterine, uterine fibroids, vignettes, clinical vignettes

## Abstract

**Background:**

Reproductive health conditions such as endometriosis, uterine fibroids, and polycystic ovary syndrome (PCOS) affect a large proportion of women and people who menstruate worldwide. Prevalence estimates for these conditions range from 5% to 40% of women of reproductive age. Long diagnostic delays, up to 12 years, are common and contribute to health complications and increased health care costs. Symptom checker apps provide users with information and tools to better understand their symptoms and thus have the potential to reduce the time to diagnosis for reproductive health conditions.

**Objective:**

This study aimed to evaluate the agreement between clinicians and 3 symptom checkers (developed by Flo Health UK Limited) in assessing symptoms of endometriosis, uterine fibroids, and PCOS using vignettes. We also aimed to present a robust example of vignette case creation, review, and classification in the context of predeployment testing and validation of digital health symptom checker tools.

**Methods:**

Independent general practitioners were recruited to create clinical case vignettes of simulated users for the purpose of testing each condition symptom checker; vignettes created for each condition contained a mixture of condition-positive and condition-negative outcomes. A second panel of general practitioners then reviewed, approved, and modified (if necessary) each vignette. A third group of general practitioners reviewed each vignette case and designated a final classification. Vignettes were then entered into the symptom checkers by a fourth, different group of general practitioners. The outcomes of each symptom checker were then compared with the final classification of each vignette to produce accuracy metrics including percent agreement, sensitivity, specificity, positive predictive value, and negative predictive value.

**Results:**

A total of 24 cases were created per condition. Overall, exact matches between the vignette general practitioner classification and the symptom checker outcome were 83% (n=20) for endometriosis, 83% (n=20) for uterine fibroids, and 88% (n=21) for PCOS. For each symptom checker, sensitivity was reported as 81.8% for endometriosis, 84.6% for uterine fibroids, and 100% for PCOS; specificity was reported as 84.6% for endometriosis, 81.8% for uterine fibroids, and 75% for PCOS; positive predictive value was reported as 81.8% for endometriosis, 84.6% for uterine fibroids, 80% for PCOS; and negative predictive value was reported as 84.6% for endometriosis, 81.8% for uterine fibroids, and 100% for PCOS.

**Conclusions:**

The single-condition symptom checkers have high levels of agreement with general practitioner classification for endometriosis, uterine fibroids, and PCOS. Given long delays in diagnosis for many reproductive health conditions, which lead to increased medical costs and potential health complications for individuals and health care providers, innovative health apps and symptom checkers hold the potential to improve care pathways.

## Introduction

### Background

Millions of women and people who menstruate worldwide are affected by reproductive health conditions. Endometriosis, uterine fibroids, and polycystic ovary syndrome (PCOS) are among the most common with prevalences estimated at 10%-15%, 20%-40%, and 5%-20%, respectively [1-12]. All 3 conditions have been associated with fertility issues [12-14]. Endometriosis is a condition where endometrial tissue is found outside of the uterus and is typically characterized by painful periods, abnormal bleeding, and chronic pelvic pain, among other symptoms [5,14,15]. Uterine fibroids are benign uterine tumors that can cause a variety of debilitating symptoms, such as heavy menstrual bleeding, pain, and bladder or bowel dysfunction [12,16]. PCOS is a complex endocrine disorder characterized by a variety of symptoms, such as menstrual dysfunction and hirsutism, of differing severity and without a certain etiology [13]. These conditions can have similar presentations; for example, both pain and intermenstrual bleeding are symptoms of endometriosis and uterine fibroids, and additionally, these conditions can coexist in individuals at the same time.

Long diagnostic delays are common for endometriosis, uterine fibroids, and PCOS, with patients reporting receiving a diagnosis between 2 and 12 years from the onset of symptoms [17-22]. Controversy over diagnostic criteria may further complicate or delay final diagnosis [12,23-25]. Another contributing factor to diagnostic delays is a low level of knowledge on reproductive health as affected persons may believe symptoms are normal or hereditary, thus delaying in seeking medical input until symptoms worsen [26]. Delays in diagnosis can lead to worsening of symptoms, further health complications with fertility or psychiatric conditions, and a reduced quality of life [26-31]. Both endometriosis and uterine fibroids severely affect quality of life, everyday functioning, and workplace productivity [32-36]. A common sequela of PCOS is also a lowered quality of life [37] but also includes infertility, type 2 diabetes, and cardiovascular and psychiatric conditions (eg, hypertension, depression, and anxiety) [38].

In addition to risks of developing complications with fertility or psychiatric conditions [27-30], long diagnostic delays are associated with increased health care use and costs [39]. Endometriosis costs an average of US $27,855 per patient annually in the United States alone [40], while overall yearly expenditure for uterine fibroids is estimated to be US $34.4 billion [35]. Further, patients with long diagnostic delays for endometriosis have 60% higher mean all-cause costs compared to those with short delays [39]. Similarly, the economic costs of PCOS on individuals and health care systems are estimated to be US $8 billion per year [8]. As diagnostic costs represent a small proportion of the total economic burden of disease, particularly in light of long diagnostic delays, access to simpler screening processes may be a cost-effective strategy [41].

Innovations in health technologies and mobile apps have the potential to bridge this economic gap, deliver better health outcomes, and improve quality of life. Worldwide, there are more than 6 billion smartphone subscribers [42] and more than 350,000 health-related mobile apps [43]. As such, people increasingly turn to the internet for health information [44-46], with an increasing number of digital health interventions existing to assist with condition diagnosis (eg, check user symptoms against common condition symptoms) [47,48].

Despite the widespread availability and advantages of symptom checker apps, there remains a knowledge gap on the accuracy of many of these tools [49]. Researchers, clinicians, and patient groups are increasingly demanding more rigorous validation and evaluation of digital health solutions, with scientists highlighting the need for evidence generation [50-53]. Case vignette studies represent an established methodology for the evaluation of digital symptom checkers. In such studies, relevant fictitious patient cases are assessed by the symptom checker under investigation, and the output is compared to that of a human expert assessing the same case [54]. However, several scoping reviews have identified significant variability in study designs and reported quantitative measures when assessing symptom checkers, with about half reports describing app characteristics and half examining actual accuracy metrics, which were found to vary greatly [49,55]*.* A recent review of digital and web-based symptom checkers found diagnostic accuracy of the primary diagnosis varied from 19% to 38% and triage accuracy ranged from 49% to 90% [56]. Even though information on their development and validation is limited and its reliability is in question [47,49], trust in symptom checker apps is high among laypersons [57].

### Flo App and Symptom Checker Development

Flo (by Flo Health UK Limited) is a health and well-being mobile app and period tracker for women and people who menstruate, with over 58 million monthly active users [58]. Flo allows users to track their symptoms throughout their menstrual cycle (eg, cramps, menstrual flow, and mood) or pregnancy and postpartum (eg, lochia), as well as general health information like contraceptive use, ovulation or pregnancy test results, water intake, and sleep. Additionally, the app offers personalized, evidence-based, and expert-reviewed content via an in-app library. Further, digital health assistants (chatbots) provide users with information about a range of conditions.

Flo has developed 3 single-condition symptom checker “chatbots” to assess symptoms of reproductive health conditions (endometriosis, uterine fibroids, and PCOS). The decision to focus on these conditions was based on their prevalence, the feasibility of symptom assessment via an app, and the multifactorial impact that these conditions can have (eg, quality of life, productivity, cardiovascular diseases, mental health conditions, and fertility). The symptom checkers use symptom information gained through conversation-like questions and answers as well as symptom or menstrual cycle information previously entered into the app. Users with acute presentations are provided with a list of red flag symptoms (eg, nausea with vomiting, fever, and vaginal bleeding not related to the period) at the beginning of the conversation and are advised to discontinue the conversation with the symptom checker and seek urgent medical advice if their presence is confirmed by the user. After the conversation, the symptom checker gives the user one of two possible outcomes: (1) a strong match for the condition—”You’re experiencing several symptoms typically associated with [condition]” or (2) a weak or no match for the condition—”While you may be experiencing some symptoms of [condition], your combination of symptoms does not strongly indicate it.” An informative summary is available for the user that reiterates which of the user’s symptoms match the presentation of a particular condition as described in the relevant clinical guidelines. This summary can then be used by the user to facilitate any subsequent conversations with their health care provider. The symptom checker is not intended as a diagnostic tool, does not provide medical advice, and users are advised to seek medical input to further investigate any concerns they have.

To ensure medical accuracy and safety during the development of symptom checkers, Flo uses a combination of an in-house medical team and external doctors specializing in the conditions of interest. The medical team builds the chat sequences considering the most relevant signs and symptoms based on the latest medical guidelines and evidence. The chat sequence is medically tested, reviewed, and adjusted in an iterative product development process.

The aim of this study was to determine the accuracy (agreement between clinician and symptom checker) of 3 symptom checkers for endometriosis, uterine fibroids, and PCOS developed using current medical guidelines (Monash, European Society of Human Reproduction and Embryology, and American Academy of Family Physicians) [24,59,60]. To this end, we devised a case vignette study whereby fictional patient cases were assessed for symptoms of the earlier-mentioned conditions by both symptom checkers and medical practitioners. We also aimed to provide a comprehensive illustration of how we created, reviewed, and categorized vignette cases in the predeployment testing and validation of digital health symptom checker tools.

## Methods

### Vignette Testing

#### Overview

Clinical case vignettes were created, reviewed, approved, classified, and entered into the symptom checkers by independent general practitioners recruited specifically for this study. Vignette cases needed to encompass presentations of not just endometriosis, fibroids, and PCOS but also other similarly presenting reproductive (eg, amenorrhea) and general health (eg, thyroid disorder) conditions. General practitioners have knowledge of a wide range of condition symptomatology and are typically the first point of contact for a patient in a health care system in the United Kingdom (where the study took place). Therefore, we reasoned that general practitioners were a more suitable choice for vignette creation, review, and classification instead of obstetricians and gynecologists.

All general practitioners were UK-based with an average of 12 years of clinical experience and were not previously affiliated with Flo. All general practitioners were remunerated for their time. No human subjects, interviews, or patient-doctor transcripts were used in the creation of vignettes; all case vignettes involved in this study are fictitious and were created from each general practitioner’s experience of treating patients with these conditions.

#### Vignette Creation, Review, and Approval

**Figure 1:**
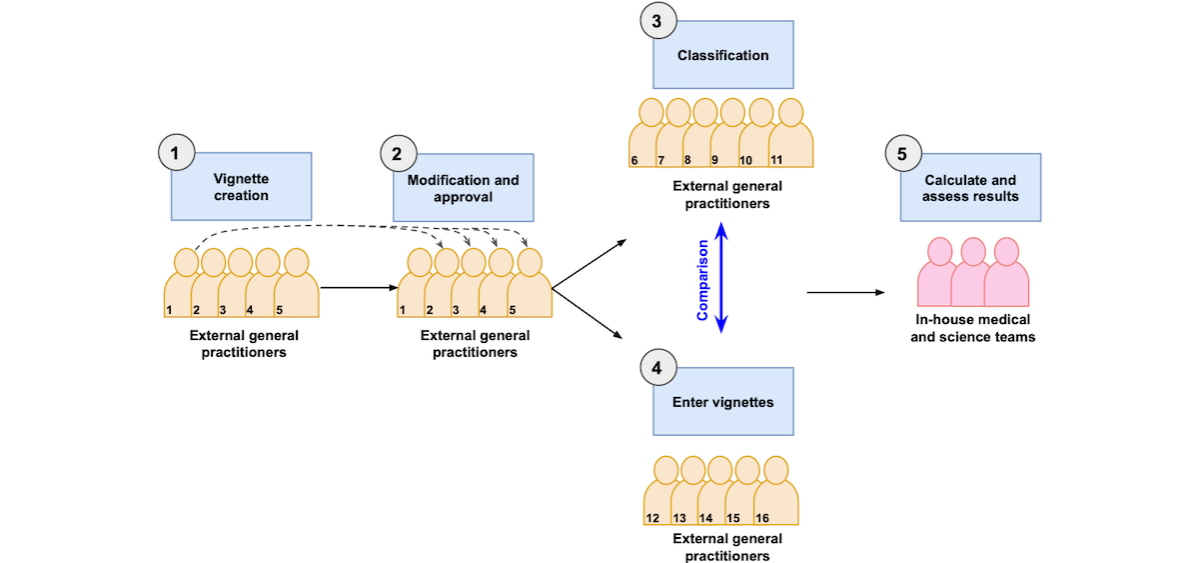
Vignette study procedure including (1) independent vignette creation by 5 external general practitioners; (2) vignette review, modification, and approval by a second and third general practitioner where required; (3) independent vignette classification by 6 external general practitioners not involved in other stages; (4) entry of vignettes into symptom checkers by 5 external general practitioners not involved in other stages; and (5) analysis of results.

Five external general practitioners were recruited to independently create clinical case vignettes of simulated users (Figure 1, step 1). These simulated users would be presenting for the first time without any history of diagnosis or treatment for 1 of the 3 conditions of interest, namely, endometriosis, uterine fibroids, or PCOS. Cases were derived from the general practitioners’ clinical experience and the literature. The general practitioners completed a template (Multimedia Appendix 1) for each vignette that contained information on the user’s background, history of presenting condition, medical, surgical, and family history, as well as details on their menstrual cycle and other symptoms. The general practitioners were instructed to create a set number of cases for each of the 3 conditions and for each of the 3 possible outcomes to ensure a spread of severity and condition types: (A) “You’re experiencing specific signs and symptoms commonly associated with [condition]”, (B) “Although you’re experiencing some of the potential signs and symptoms of [condition], they are not specific enough to indicate it strongly,” and (C) “You’re not experiencing any of the signs and symptoms commonly associated with [condition].” The general practitioners were instructed that “A” cases are those for which the user has specific features of the condition, and this differential diagnosis is the most likely cause of their symptoms. “B” and “C” cases are those which are considered to not have the condition. General practitioners were instructed that “B” cases represent users who show either too few or only some specific fındings, and a clinician would not think of this condition as the most likely cause for these symptoms. “C” cases represent users who show either too few or nonspecific symptoms, and there would be other differential diagnoses that are more likely to be the cause of the symptoms. Condition-negative cases had other diagnoses such as urinary tract infection, thrush, pregnancy, and functional constipation.

Each vignette was reviewed by a second general practitioner (Figure 1, step 2) who could either approve the vignette as-is or suggest changes to clarify the case. If changes were suggested, the case would then be reviewed, edited, and approved by a third general practitioner who would finalize the case. In total, 24 cases were created for each condition, in line with other single-condition or single-system symptom checker evaluations [61-64].

#### Independent Classification of Vignettes

After vignette approval (Figure 1, step 2), all information related to the intended designation of each vignette was removed: the type of case (A, B, or C above) and any notes about the diagnosis the creator had in mind when creating the vignette were removed from the vignette template. To avoid bias from the case creator when setting the final classification, an additional independent panel of 6 additional external general practitioners not involved in previous steps of the vignette creation was recruited to classify the vignettes (Figure 1, step 3). The classifying general practitioners received a random selection of vignettes, each designated as either an endometriosis vignette, uterine fibroid vignette, or PCOS vignette. For each vignette, the general practitioners reviewed the case and designated the most likely outcome for the specified condition (endometriosis, uterine fibroids, or PCOS) matching the symptom checker wording: (1) a strong match for the condition—“You’re experiencing several symptoms typically associated with [condition]” or (2) weak or no match for the condition—“While you may be experiencing some symptoms of [condition], your combination of symptoms does not strongly indicate it.” During this step of classification, to ensure there was a shared agreement on the classification of each case, each vignette was reviewed independently by 3 general practitioners; the majority view (at least 2 out of 3) was taken as the “true value” or gold-standard classification for the vignette. While the vignettes were created with 3 levels of categorization for each condition, the classifying general practitioners were not aware of these levels and were asked to make a binary classification for each vignette.

#### Vignette Entry

An additional set of 5 external general practitioners (not involved in the other steps) were recruited to enter the vignette cases into a prototype of the symptom checkers (Figure 1, step 4). At this stage, the general practitioners were blinded to the condition assigned to the vignette, the classification, and the condition the symptom checker was assessing. If the symptom checker asked a question that was not contained in the vignette, general practitioners were instructed to follow a step-by-step protocol to determine the appropriate answer. First, if the symptom information requested by the symptom checker was specified in the vignette template but not included by the creator, a negative response should be selected (eg, the vignette template specifies pain symptoms should include whether the radiation of pain is present, but the vignette creator does not detail this in their description of pain, then pain radiation should be assumed to be absent). If the information was not part of the template, a neutral response (eg, “I don’t know” and “I don’t want to answer this question”) should be selected. If no neutral response was available, a negative response should be selected. If no negative response was available, the answer mostly within normal limits should be selected (eg, the inputting general practitioner would select a period length of 2-7 days, as opposed to a period length of 1 day or less or a period length of 8 days or more).

### Analysis

**Figure 2:**
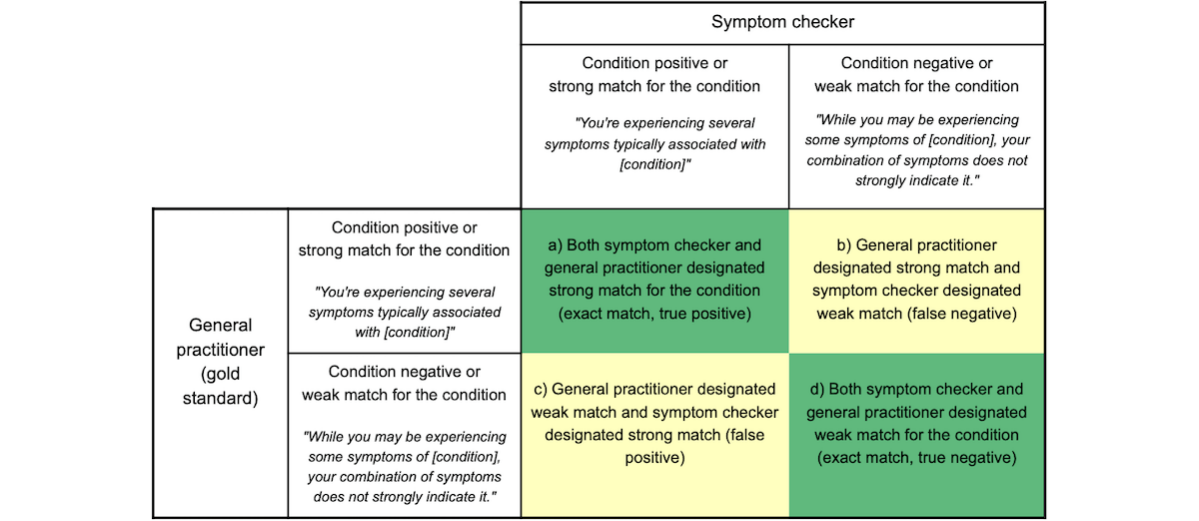
Two-way validation table demonstrating the true positive, true negative, false positive, and false negative cases produced when comparing the symptom checker output to the general practitioner gold standard.

The final classification set by the independent general practitioner classifiers (Figure 1, step 3) was compared with the outcome of the symptom checker as tested in Figure 1, step 4. Outcomes were arranged in 2-way tables as shown in Figure 2. Accuracy statistics for percent agreement between general practitioner classification and symptom checker, sensitivity, specificity, positive predictive value (PPV), and negative predictive value (NPV) were calculated using the true positives (TP), true negatives (TN), false positives (FP), and false negatives (FN) as detailed: accuracy (percent agreement): (TP+TN)/(TP+TN+FP+FN); sensitivity: TP/(TP+FN); specificity: TN/(FP+TN); PPV: TP/(TP+FP); and NPV: TN/(FN+TN) (Figure 2).

## Results

### Vignette Cases

Of the total of 24 cases that were created per condition (Table 1), 11-13 cases were classified as a strong match for the condition, and 11-13 cases were classified as a weak match for the condition after final classification by a panel (shown in Figure 1, step 3).

**Table 1:** Two-way validation table by condition (endometriosis [E], uterine fibroids [UF], and polycystic ovary syndrome [P]).

			Condition positive or strong match for the condition						Condition negative or weak match for the condition							Total				
			E, n		UF, n		P, n	E, n			UF, n		P, n		E, n			UF, n		P, n
**General practitioner (gold standard)**																				
	Condition positive or strong match for the condition	9		11		12		2		2		0		11			13		12	
	Condition negative or weak match for the condition	2		2		3		11		9		9		13			11		12	
Total			11		13		15	13			11		9		24			24		24

### Accuracy Metrics

**Figure 3:**
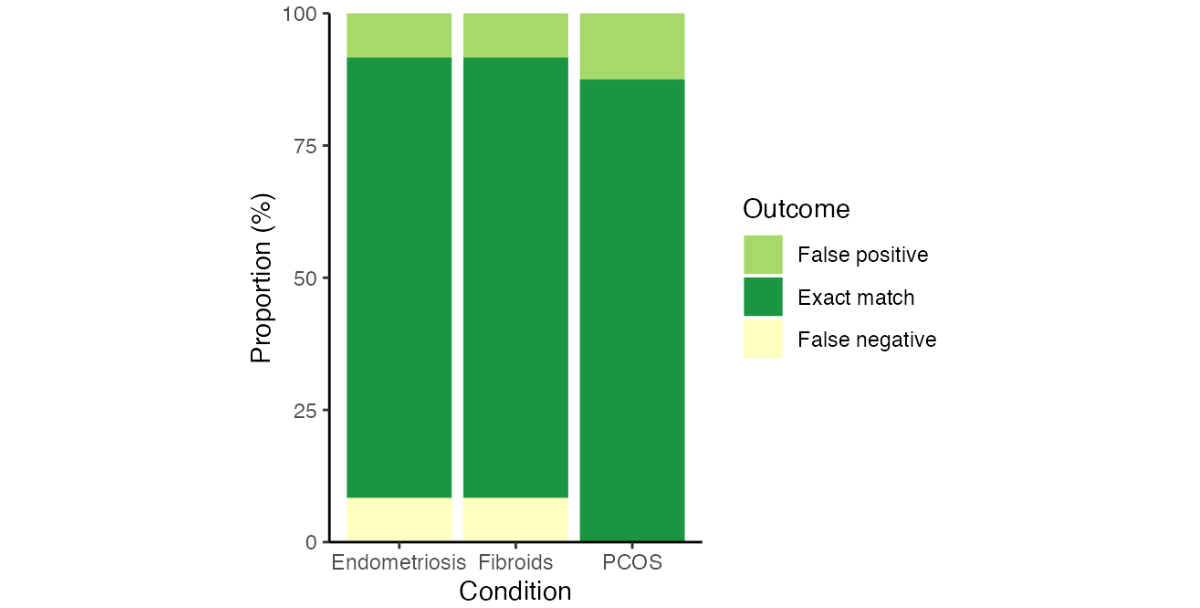
Overall symptom checker performance showing the proportion of false-positive outcomes, exact match outcomes, and false-negative outcomes by condition. PCOS: polycystic ovary syndrome.

Overall, exact matches (percent agreement) between the vignette classification and the symptom checker outcome ranged from 83% (20/24) for endometriosis and uterine fibroids to 88% (21/24) for PCOS (Figure 3 and Table 2). While there were no FN outcomes for PCOS, 8% (6/72) of all cases were falsely identified by the relevant symptom checker as negative for endometriosis and uterine fibroids. FP outcomes ranged from 8% (2/24) for endometriosis and uterine fibroids to 13% (3/24) of all cases for PCOS. An example vignette case showing a TP, TN, FP, and FN case (determined by agreement between general practitioner and symptom checker) is provided for each condition in Multimedia Appendix 2.

**Table 2:** Accuracy metrics for endometriosis, fibroids, and polycystic ovary syndrome (PCOS).

Condition	Values, n	Agreement (%)	Sensitivity (%)	Specificity (%)	PPV^a^ (%)	NPV^b^ (%)
Endometriosis	24	83.3	81.8	84.6	81.8	84.6
Fibroids	24	83.3	84.6	81.8	84.6	81.8
PCOS	24	87.5	100	75	80	100

^a^PPV: positive predictive value.

^b^NPV: negative predictive value.

While sensitivity was very high (100%) for PCOS (Table 2), specificity was high for all 3 conditions (>81%). PPV ranged from 80% for PCOS to 84.6% for uterine fibroids, while NPV ranged from 81.8% for uterine fibroids to 100% for PCOS.

## Discussion

### Summary

In this study, we provide an example methodology for the creation, review, and classification of vignette cases for the testing and validation of digital health symptom checker tools. The percent agreement between general practitioner-designated vignette cases and 3 single-condition symptom checkers for 3 reproductive health conditions (endometriosis, fibroids, and PCOS) was assessed. We found the designation given to case vignettes by the symptom checkers had high levels of agreement between general practitioner and symptom checker (83.3%-87.5%), sensitivity (81.8%-100%), specificity (75%-84.6%), PPV (80%-84.6%), and NPV (81.8%-100%) when compared to gold standard designation by general practitioners. Overall, these metrics show the high performance of the symptom checkers when tested on robustly designed clinical vignettes.

### Comparison With Prior Work

This high accuracy of the identification of reproductive health conditions is particularly important as high rates of diagnostic error are reported by patients. A study of patients with self-reported surgically confirmed endometriosis found that 75.2% of patients reported being misdiagnosed with another physical health or mental health problem by their health care professional [65]. A similar study of patients diagnosed with PCOS found that 33.6% of women reported >2 years time to diagnosis, 47.1% visited ≥3 health professionals before a diagnosis was established, and 64.8% were dissatisfied with the diagnostic process [66]. The use of a tool like a symptom checker could give the user better knowledge and awareness of their symptoms in conversations they may have with their health care provider, leading to a more effective diagnostic pathway. We have shown in previous research that users agree Flo increases their knowledge of the menstrual cycle and facilitates easier conversations with their health care provider [67].

Other vignette studies of multicondition symptom checkers have shown mixed results for accuracy. A study by Gilbert et al [68] comparing urgency advice (ie, triage) from 7 multicondition symptom checker apps and 7 general practitioners to gold-standard vignettes found that the condition suggested first matched the gold standard (ie, M1 accuracy) for 71% of general practitioners and 26% of apps; when broadening to the condition suggested in the top 5 (ie, M5 accuracy), the accuracy of general practitioners rose to 83% and apps to 41%. Another study by Schmieding et al [69] comparing 22 symptom checkers using 45 vignettes found M1 accuracy of 46%, and M10 accuracy was 71%.

The multicondition symptom checkers evaluated by Gilbert et al [68] and Schmieding et al [69] were assessed using vignette cases that covered both common and less-common conditions seen in primary care practice, conditions that affect all body systems, and conditions that have a range of urgency levels. Further, these evaluated symptom checkers are designed to detect a wide range of conditions for a general population. In contrast, this study evaluated single-condition symptom checkers using vignettes specifically designed to represent presentations with specific symptoms of the condition (strong match or condition positive) and presentations with symptoms not specific to the condition (weak match or condition negative). This symptom checker design difference may explain the variation in accuracy found between our symptom checkers (single condition) and other studied symptom checkers (multicondition).

Evaluations of single-condition symptom checkers include a study of 12 web-based symptom checkers for COVID-19 [70] and a study of an app-based symptom checker for PCOS [62]. COVID-19 symptom checkers ranged widely in both sensitivity (14%-94%) and specificity (29%-100%), with only 4 symptom checkers having both sensitivity and specificity above 50% and 2 with both sensitivity and specificity above 75%. Sensitivity and specificity in our symptom checkers were between 75% and 100%. The PCOS symptom checker evaluated by Rodriguez et al [62] reported 12%-25% FP cases and no FN out of 8 cases tested. Our PCOS symptom checker had no FNs and 3 (13%) FP cases out of 24 cases tested. Our PPV and NPV values were 80%-100% for our 3 symptom checkers, suggesting relatively high chances that positively tested cases truly have the condition in question.

With the exception of COVID-19, which has a symptomatology and overall presentation that differs greatly from the reproductive health disorders assessed in this study, digital or app-based symptom checkers for a single condition are uncommon. Symptom-based patient-completed questionnaires and screening tools do exist, including for common reproductive health conditions such as endometriosis or PCOS. A patient self-assessment tool for endometriosis with 21 questions found sensitivity of 76% and specificity of 72%, PPV of 73%, and NPV of 75% [71]. Our endometriosis symptom checker had a similar but slightly higher sensitivity (81.8%), specificity (84.6%), PPV (81.8%), and NPV (84.6%). A 4-item questionnaire for use in the diagnosis of PCOS among women with a primary complaint of infertility had 77% sensitivity and 94% specificity, and a PPV and NPV calculated from their data as 87% and 88%, respectively [72]. Our PCOS symptom checker had higher sensitivity (100%), lower specificity (75%), higher NPV (100%), and lower PPV (80%), prioritizing the identification of cases. It should be noted, however, that our symptom checker is designed to be for a broader population than the 4-item clinical tool, including those who are not trying to get pregnant or experiencing fertility issues. Questionnaires such as these have some limitations. They may not be available to the public and additionally may be subject to more user error (eg, question skipping). App-based symptom checkers, on the other hand, can use historical data from users such as menstrual regularity to improve the accuracy of user answers. Additionally, users cannot accidentally skip questions, and the app will provide a detailed summary of results and recommendations.

It is not uncommon for variation of opinion between groups of general practitioners reviewing vignettes with El-Osta et al [73] reporting classification agreement between 3 general practitioners’ primary diagnosis and the intention of the vignette being 32.4%. Each vignette in this study was reviewed independently by 3 different general practitioners, and in 71% (51/72) of cases, all 3 general practitioners agreed with the vignette assignment given at the vignette approval stage (Figure 1, step 2). However, it should be noted that El-Osta et al [73] provided a comparison between primary diagnosis of general practitioners and original vignette intention, whereas this analysis only concerns strong or weak match for known reproductive conditions. All 3 general practitioners agreed with each other (regardless of the vignette intention) for 81% (58/72) of vignette cases. This disagreement between general practitioners and some differences with the symptom checker results are to be expected, particularly when using symptom-based assessment for reproductive health conditions that can be complicated to diagnose, have overlapping symptomatology with other system conditions such as gastrointestinal and urinary conditions, and are often dismissed or considered to be “normal” variations in the menstrual cycle by some. These conditions have a notoriously prolonged time to diagnosis [16-19] and require investigations including imaging. Further, the sensitivity of different testing methods can vary. For example, physical examination for deep infiltrating endometriosis can have poor accuracy and requires imaging [74].

The possible applications of symptom checkers and health apps are far-reaching and could have benefits at the individual user level, health care professional level, and macro or health system level [63,75]. Especially for many reproductive health conditions where the time to diagnosis is currently long and contributes to high health care costs [17,26,66,76], an earlier diagnosis can lead to early treatment and thus decrease complications from untreated conditions and decrease health care costs of treating more advanced disease [27,28,39]. Menstrual cycle details such as cycle length, period length, or flow can be important information for health care providers when diagnosing patients. Health apps can help track cycle details over time and use these details when determining risk for conditions as well as in summary information for users to share with their health care providers (eg, the Flo app provides a “health report” where you can download a summary of symptoms over a period of time, average cycle length, and other details to share with a health care provider). Additionally, as people with symptoms such as heavy bleeding or menstrual pain may believe these are normal or hereditary [26], personalized assessment of symptoms and encouragement to seek further evaluation from a medical professional where appropriate may improve an individual’s understanding of their symptoms and health status and decrease time to diagnosis. Our prior research has demonstrated that 58% of Flo users report improvements in understanding the normality of certain cycle-related symptoms and recognizing the abnormal nature of others, while 1 in 3 Flo users reported that the use of the Flo app improved their communication with their health care provider [67]. Therefore, mobile apps with symptom checkers could identify users with risk factors for certain conditions, educate users about their symptoms, and further encourage conversation with their medical providers.

### Strengths and Limitations

Strengths of this study include the use of different groups of independent, external general practitioners unfamiliar with the symptom checkers to create, enter, and classify case vignettes for symptom checker testing. Additionally, vignettes were created with a wide range of symptomatology to ensure the inclusion of borderline presentations as these are notoriously difficult to assess, even for doctors, although they represent a frequent reality as people do not often fit neatly into textbook case presentations. Further, each vignette case was reviewed by an independent, experienced general practitioner and classified by a separate panel viewing the vignettes for the first time. When generating vignette cases that represent typical presentations of a single condition as seen by a general practitioner, there will only be so many permutations of symptomatology that can be generated before repetitions of vignette cases occur; as a result, we created 72 vignette cases in total, 24 for each of our 3 conditions. The number of vignettes needed to evaluate symptom checkers is not well defined [54]. Other vignette symptom checker evaluations have used between 3 and 400 cases for testing, with single-condition or single-system evaluations (eg, mental health, ophthalmology, and PCOS) using fewer cases and multicondition evaluations using larger numbers of cases [61-63,77,78]. Among the 400 vignettes published by Hammoud et al [78], any single condition is only represented by at most 5 cases.

Limitations, however, should be noted. Vignette studies rely on clinical opinion of a small number of general practitioners. An audit study of clinical vignette benchmarking has shown significant variation between groups of general practitioners considering clinical vignettes [73]. To decrease bias from differences in clinical opinion, all cases were blindly reviewed by 3 general practitioners, one-third involved in cases of disagreement. We found agreement between all 3 general practitioners in 81% (58/72) of our cases. Vignettes also rely on the classical presentation of conditions that may present differently in real life or in patients with complex or atypical condition presentations. When creating vignettes, we recognize there is a possibility for bias, expected patterns, or entrenched unknowns in the understanding of each condition’s symptomatology. Additionally, although we recognize that patients do not usually present to primary care practitioners with a prespecified suspected diagnosis and that therefore this aspect of the study design does not reflect usual medical practice, these chatbots are not meant to replace the interaction with primary care providers but rather to allow users to review their symptoms in advance of seeing a health care professional. As outlined in the medical guidelines, symptom severity, risk, and prevalence may vary across world regions and ethnicities. Neither the Flo app nor the symptom checker collect data on the user’s race or ethnicity, so neither race nor ethnicity were included in the vignette creation process. In addition to this, the vignettes in this study were created by panels of general practitioners based in the United Kingdom and may not provide an accurate representation of symptoms for every cultural context. We recognize the inclusion of such information could help to identify at-risk individuals better.

While we found 100% sensitivity for our PCOS symptom checker, it is likely with a larger sample size and real-life cases, this level of perfect sensitivity will not be maintained. Other changes in accuracy statistics are likely to be seen in real-world use. Further, as real-world users may interpret their symptoms and the questions differently than doctors, future studies including the general population should be carried out to test each symptom checker’s performance in the context of real-world deployment. The use of vignette-patient cases is an important part of predeployment algorithm testing for digital symptom checkers [54,79] and is the first stage of our symptom checker evaluation. The next stage in evaluating our symptom checkers will include the use of real-world data such as observational studies of condition diagnosed and undiagnosed people’s symptoms and early field-testing of the in-app symptom checkers on users and comparing the output to an official diagnosis from a doctor. Evaluation of symptom checkers and digital health tools should follow multistage processes with increasing exposure to real environments exploring not only effectiveness but also usability and balance between probability of disease and risk of missing a diagnosis [79].

### Conclusions

In conclusion, we have described a methodology for creating and classifying vignettes using multiple independent panels of general practitioners for the predeployment testing of digital health symptom checker tools. We found high levels of agreement between general practitioner classification and single-condition symptom checkers for 3 reproductive health conditions (endometriosis, fibroids, and PCOS). Given long delays in diagnosis for many reproductive health conditions, which lead to increased medical costs and potential health complications, innovative health apps and symptom checkers hold the potential to improve care pathways.

## Appendix

Multimedia Appendix 1 Vignette template.

Multimedia Appendix 2 Example case vignettes with matching and mismatching classification between general practitioners and the symptom checker (SC).

## Abbreviations

FN: false negative
FP: false positive
NPV: negative predictive value
PCOS: polycystic ovary syndrome
PPV: positive predictive value
TN: true negative
TP: true positive
