# Supplementary material for: Assessment of a Digital Symptom Checker Tool's Accuracy in Suggesting Reproductive Health Conditions: Clinical Vignettes Study
Source: JMIR Mhealth Uhealth. 2023 Dec 5;11:e46718. doi: 10.2196/46718 (PMC10731551; doi:10.2196/46718)
Supplement: Multimedia Appendix 1 [file mhealth_v11i1e46718_app1.docx]

## **Multimedia Appendix 1.** Vignette template.

|  | **Stage 1 (creation)**  Write the complete vignette in the following boxes. Do not leave any boxes blank, use “none” or “not applicable” if needed. | **Stage 2.1 (review)**  If additional information is needed to distinguish between:   1. *“You're experiencing specific signs and symptoms commonly associated with [condition]”* 2. *"Although you're experiencing some of the potential signs and symptoms of [condition], they are not specific enough to indicate it strongly."* 3. *"You're not experiencing any of the signs and symptoms commonly associated with [condition]."*   Please put suggestions in the following boxes. | **Stage 2.2 (approval)**  If any additions or changes are suggested in stage 2.1, a third GP should review and approve the final vignette. Please use the following boxes to record the final vignette. Do not leave any boxes blank, use “none” or “not applicable” if needed. |
| --- | --- | --- | --- |
| **GP ID:** |  |  |  |
|  |  |  |  |
| **Condition assigned** |  |  |  |
| **Likelihood of condition** |  |  |  |
| **Actual simulated condition you have in mind** |  |  |  |
|  |  |  |  |
| **Age** |  |  |  |
| **Sex** |  |  |  |
| **BMI** |  |  |  |
| **Smoking status** |  |  |  |
| **Alcohol intake (units per week)** |  |  |  |
| **Medication** |  |  |  |
| **LMP** |  |  |  |
| **Gravidity** |  |  |  |
| **Parity** |  |  |  |
| **Chief complaints** |  |  |  |
| **History of presenting illness**  (please indicate for all symptoms: duration and frequency. Specifically for pain, please include Site, Onset, Character, Radiation, Associations, Time course (e.g. cyclical nature or pattern), Exacerbating/ relieving factors, severity (e.g. mild, moderate, severe, extremely severe) |  |  |  |
| **Absent findings** |  |  |  |
| **Past medical and surgical history** |  |  |  |
| **Menstrual cycle length and regularity** | Average cycle length:  Cycle regularity (number of days difference between shortest and longest cycle in the past year):  Average period length:  Bleeding volume during period (low/medium/heavy, if heavy, is it ever enough to soak more than one tampon/pad every hour several hours in a row, clots):  Any missed periods/periods of amenorrhea: |  |  |
| **Menstrual pain or problems**  (e.g. any bleeding outside of period, any bloating or constipation and if it’s related to period timing, any association with bowel movements, urination, sex. For pain please include Site, Onset, Character, Radiation, Associations, Time course (e.g. cyclical nature or pattern), Exacerbating/ relieving factors) |  |  |  |
| **Menstrual pain severity/frequency** (if applicable)  *Check the relevant box* | - No menstrual pain   Severity:   - Not applicable - Mild - Moderate - Severe - Extremely severe   Frequency:   - Never - Sometimes (once every 2-3 cycles) - Regularly (A few days every cycle) - Always (every cycle, almost all the time) |  |  |
| **Obstetric history**  (include time spent trying to conceive, if applicable) |  |  |  |
| **Gynae history**  (please include vaginal discharge characteristics if applicable including vaginal dryness) |  |  |  |
| **Sexual history**  (include post-coital vaginal bleeding, dyspareunia - on initial penetration, deep penetration or both, vaginal dryness, changes in libido, or other sexual or contraception concerns) |  |  |  |
| **Family history** |  |  |  |
| **Any additional information**  (e.g. regularly bothered by gastrointestinal, urinary, mental/emotional issues, fatigue, sleep disturbances, changes in appetite or eating, skin changes - severe acne, hyperpigmentation, baldness, hirsutism (including location)) |  |  |  |
| **Impact of bleeding and pain on quality of life (if applicable)**  *Check the relevant box* | - No bleeding/pain   Frequency:   - No impact on quality of life - Sometimes (at least once per month, or once per 2-3 cycles) - Regularly (at least once per week, or a few days per cycle) - Always (almost every day, or every cycle almost all of the time) |  |  |
|  |  | - tick if approving the original vignette with no changes needed | If reviewed by a third GP, please ensure all information for the final vignette is included in this column, no other columns will be considered. |

## 
