# Supplementary material for: Assessment of a Digital Symptom Checker Tool's Accuracy in Suggesting Reproductive Health Conditions: Clinical Vignettes Study
Source: JMIR Mhealth Uhealth. 2023 Dec 5;11:e46718. doi: 10.2196/46718 (PMC10731551; doi:10.2196/46718)
Supplement: Multimedia Appendix 2 [file mhealth_v11i1e46718_app2.docx]

## **Multimedia Appendix 2.** Example vignettes cases with matching and mismatching classification between general practitioners and symptom checker (SC).

### Endometriosis vignette cases: True positive, True negative, False negative, False positive

| **Condition** | Endometriosis |
| --- | --- |
| **GP classification** | Strong match |
| **SC classification** | Strong match |
| **Classification agreement** | True positive |
|  |  |
| **Age** | 19 |
| **Sex** | Female |
| **BMI** | 19 |
| **Smoking status** | Smokes 10 cigarettes a day |
| **Alcohol intake (units per week)** | 10 |
| **Medication** | Combined contraceptive pill  Cetraban emollient  Vitamin D cream  Eumovate cream |
| **LMP** | 1 week ago |
| **Gravidity** | 0 |
| **Parity** | 0 |
| **Chief complaints** | Chronic pelvic pain  Painful sexual intercourse |
| **History of presenting illness**  (please indicate for all symptoms: duration and frequency. Specifically for pain, please include Site, Onset, Character, Radiation, Associations, Time course (e.g. cyclical nature or pattern), Exacerbating/ relieving factors, severity (e.g. mild, moderate, severe, extremely severe) | Constant pain in the pelvis for the last 8 months. Worse during period. Severe and aching pain. No radiation.  Associated with pain opening bowels.  Deep pelvic pain on sexual intercourse. |
| **Absent findings** | None |
| **Past medical and surgical history** | Psoriasis |
| **Menstrual cycle length and regularity** | Average cycle length: 28  Cycle regularity (number of days difference between shortest and longest cycle in the past year): 1  Average period length:7  Bleeding volume during period (low/medium/heavy, if heavy, is it ever enough to soak more than one tampon/pad every hour several hours in a row, clots): medium  Any missed periods/periods of amenorrhea:No |
| **Menstrual pain or problems**  (e.g. any bleeding outside of period, any bloating or constipation and if it’s related to period timing, any association with bowel movements, urination, sex. For pain please include Site, Onset, Character, Radiation, Associations, Time course (e.g. cyclical nature or pattern), Exacerbating/ relieving factors) | Regular periods.  No intermittent bleeding or spotting.  Pelvic pain worse.  No constipation.  Painful to open bowels during periods.  Normal urine.  Menarche age 10 years old |
| **Menstrual pain severity/frequency** (if applicable)  *Check the relevant box* | No menstrual pain  Severity:  Not applicable  Mild  Moderate  Severe  Extremely severe  Frequency:  Never  Sometimes (once every 2-3 cycles)  Regularly (A few days every cycle)  Always (every cycle, almost all the time) |
| **Obstetric history**  (include time spent trying to conceive, if applicable) | Never been pregnant or tried to conceive |
| **Gynae history**  (please include vaginal discharge characteristics if applicable including vaginal dryness) | No vaginal discharge  No vaginal dryness |
| **Sexual history**  (include post-coital vaginal bleeding, dyspareunia - on initial penetration, deep penetration or both, vaginal dryness, changes in libido, or other sexual or contraception concerns) | Sexually active with regular partner - takes combined contraceptive pill.  Deep dyspareunia  No post coital bleeding  No change to libido |
| **Family history** | Mother and Maternal aunt have endometriosis |
| **Any additional information**  (e.g. regularly bothered by gastrointestinal, urinary, mental/emotional issues, fatigue, sleep disturbances, changes in appetite or eating, skin changes - severe acne, hyperpigmentation, baldness, hirsutism (including location)) | Painful opening bowels during period |
| **Impact of bleeding and pain on quality of life (if applicable)**  *Check the relevant box* | No bleeding/pain  Frequency:  No impact on quality of life  Sometimes (at least once per month, or once per 2-3 cycles)  Regularly (at least once per week, or a few days per cycle)  Always (almost every day, or every cycle almost all of the time) |

| **Condition** | Endometriosis |
| --- | --- |
| **GP classification** | Weak match |
| **SC classification** | Weak match |
| **Classification agreement** | True negative |
|  |  |
| **Age** | 22 |
| **Sex** | F |
| **BMI** | 23 |
| **Smoking status** | Vapes regularly |
| **Alcohol intake (units per week)** | 1-2 |
| **Medication** | Nil regular |
| **LMP** | 5 days ago |
| **Gravidity** | 0 |
| **Parity** | 0 |
| **Chief complaints** | Pain when passing urine |
| **History of presenting illness**  (please indicate for all symptoms: duration and frequency. Specifically for pain, please include Site, Onset, Character, Radiation, Associations, Time course (e.g. cyclical nature or pattern), Exacerbating/ relieving factors, severity (e.g. mild, moderate, severe, extremely severe) | Noticing a burning pain when passing urine for the past two days. She has a constant feeling of needing to pass urine and has been going very frequently. She has had a bit of suprapubic pain as well. She has had cystitis before and this feels the same. |
| **Absent findings** | No blood in the urine, no fever, no back pain. |
| **Past medical and surgical history** | One previous episode of cystitis |
| **Menstrual cycle length and regularity** | Average cycle length: 27 days  Cycle regularity (number of days difference between shortest and longest cycle in the past year): 3  Average period length: 6 days  Bleeding volume during period (low/medium/heavy, if heavy, is it ever enough to soak more than one tampon/pad every hour several hours in a row, clots): Low  Any missed periods/periods of amenorrhea: None |
| **Menstrual pain or problems**  (e.g. any bleeding outside of period, any bloating or constipation and if it’s related to period timing, any association with bowel movements, urination, sex. For pain please include Site, Onset, Character, Radiation, Associations, Time course (e.g. cyclical nature or pattern), Exacerbating/ relieving factors) | Regular periods, no particular issues with them. |
| **Menstrual pain severity/frequency** (if applicable)  *Check the relevant box* | No menstrual pain  Severity:   - Not applicable - Mild - Moderate - Severe - Extremely severe   Frequency:   - Never - Sometimes (once every 2-3 cycles) - Regularly (A few days every cycle) - Always (every cycle, almost all the time) |
| **Obstetric history**  (include time spent trying to conceive, if applicable) | None, never tried to conceive. |
| **Gynae history**  (please include vaginal discharge characteristics if applicable including vaginal dryness) | None. No unusual discharge, no unexpected bleeding. |
| **Sexual history**  (include post-coital vaginal bleeding, dyspareunia - on initial penetration, deep penetration or both, vaginal dryness, changes in libido, or other sexual or contraception concerns) | Occasionally sexually active with casual partners. Uses condoms for contraception every time and does not experience any pain during intercourse. Last had intercourse just before her symptoms started. |
| **Family history** | Nil significant |
| **Any additional information**  (e.g. regularly bothered by gastrointestinal, urinary, mental/emotional issues, fatigue, sleep disturbances, changes in appetite or eating, skin changes - severe acne, hyperpigmentation, baldness, hirsutism (including location)) | None |
| **Impact of bleeding and pain on quality of life (if applicable)**  *Check the relevant box* | No bleeding/pain  Frequency:   - No impact on quality of life - Sometimes (at least once per month, or once per 2-3 cycles) - Regularly (at least once per week, or a few days per cycle) - Always (almost every day, or every cycle almost all of the time) |

| **Condition** | Endometriosis |
| --- | --- |
| **GP classification** | Strong match |
| **SC classification** | Weak match |
| **Classification agreement** | False negative |
|  |  |
| **Age** | 38 |
| **Sex** | Female |
| **BMI** | 24.2 |
| **Smoking status** | Non-smoker |
| **Alcohol intake (units per week)** | 7 |
| **Medication** | Paracetamol and ibuprofen as needed  Occasional sumatriptan for migraine |
| **LMP** | 2 days ago |
| **Gravidity** | 4 |
| **Parity** | 2 |
| **Chief complaints** | Pain on defecation |
| **History of presenting illness**  (please indicate for all symptoms: duration and frequency. Specifically for pain, please include Site, Onset, Character, Radiation, Associations, Time course (e.g. cyclical nature or pattern), Exacerbating/ relieving factors, severity (e.g. mild, moderate, severe, extremely severe) | About 6 months ago started to notice occasional pain on opening bowels. Initially put this down to slight constipation, but has persisted and worsened since then.  Now finds it very painful to open bowels around the time of her period.  Sharp, stabbing pain in the rectal area.  Minimal bleeding on rare occasions - ‘one or two drops of blood’ on wiping.  Not clear if paracetamol or ibuprofen help.  Able to open her bowels pain-free at other times of the month. |
| **Absent findings** | No change to bowel habit (other than longstanding mild constipation)  Not passing any mucus PR.  No abdominal pain other than typical period pain during menstruation  No weight loss  No post-coital or intermenstrual bleeds  No unusual discharge  No other systemic symptoms |
| **Past medical and surgical history** | 2 previous miscarriages in 2014 and 2017. Both required medical management but this was straightforward in both cases.  2 live births delivered by normal vaginal delivery (with no perineal tearing) in 2015 and 2018.  Broken tibia/fibula as a teenager - fixed surgically at the time and no problems since.  Classical migraine - since teenage years. Pattern unchanged and responds well to sumatriptan. |
| **Menstrual cycle length and regularity** | Average cycle length: 30 days  Cycle regularity (number of days difference between shortest and longest cycle in the past year): 5 days  Average period length: 7 days  Bleeding volume during period (low/medium/heavy, if heavy, is it ever enough to soak more than one tampon/pad every hour several hours in a row, clots): Medium  Any missed periods/periods of amenorrhea: No |
| **Menstrual pain or problems**  (e.g. any bleeding outside of period, any bloating or constipation and if it’s related to period timing, any association with bowel movements, urination, sex. For pain please include Site, Onset, Character, Radiation, Associations, Time course (e.g. cyclical nature or pattern), Exacerbating/ relieving factors) | Many years of painful periods. Pain begins 2-3 days prior to menstruation and lasts for most of it. Described as ‘moderate’ and ‘a dull ache’, 4-5/10. Settles well with paracetamol and able to work through it.  Pain is suprapubic with occasional radiation to the upper thighs.  Wonders if it is worse when she has had more alcohol or caffeine than usual, but no clear link. |
| **Menstrual pain severity/frequency** (if applicable)  *Check the relevant box* | No menstrual pain  Severity:  Not applicable  Mild  Moderate  Severe  Extremely severe  Frequency:  Never  Sometimes (once every 2-3 cycles)  Regularly (A few days every cycle)  Always (every cycle, almost all the time) |
| **Obstetric history**  (include time spent trying to conceive, if applicable) | 4 previous pregnancies, all of which were conceived within 4 months of trying. 2 ended in spontaneous first-trimester miscarriage. 2 ended in healthy live births. No pregnancy complications or perineal trauma. |
| **Gynae history**  (please include vaginal discharge characteristics if applicable including vaginal dryness) | Nil of note. |
| **Sexual history**  (include post-coital vaginal bleeding, dyspareunia - on initial penetration, deep penetration or both, vaginal dryness, changes in libido, or other sexual or contraception concerns) | Occasional deep dyspareunia around the time of her period. Has happened 3-4 times in the past year, lasts a few seconds only.  Occasional dryness, but nil consistent. |
| **Family history** | Mum and sister both have asthma  Aunt has endometriosis  No bowel disease |
| **Any additional information**  (e.g. regularly bothered by gastrointestinal, urinary, mental/emotional issues, fatigue, sleep disturbances, changes in appetite or eating, skin changes - severe acne, hyperpigmentation, baldness, hirsutism (including location)) | No other issues |
| **Impact of bleeding and pain on quality of life (if applicable)**  *Check the relevant box* | No bleeding/pain  Frequency:  No impact on quality of life  Sometimes (at least once per month, or once per 2-3 cycles)  Regularly (at least once per week, or a few days per cycle)  Always (almost every day, or every cycle almost all of the time) |

| **Condition** | Endometriosis |
| --- | --- |
| **GP classification** | Weak match |
| **SC classification** | Strong match |
| **Classification agreement** | False positive |
|  |  |
| **Age** | 48 |
| **Sex** | F |
| **BMI** | 24 |
| **Smoking status** | Non smoker |
| **Alcohol intake (units per week)** | 10 |
| **Medication** | Nil regular |
| **LMP** | 7 days ago |
| **Gravidity** | 1 |
| **Parity** | 0 |
| **Chief complaints** | Intermenstrual bleeding |
| **History of presenting illness**  (please indicate for all symptoms: duration and frequency. Specifically for pain, please include Site, Onset, Character, Radiation, Associations, Time course (e.g. cyclical nature or pattern), Exacerbating/ relieving factors, severity (e.g. mild, moderate, severe, extremely severe) | Presenting with intermenstrual bleeding. Her periods had previously been regular, but for the past few months she’s had bleeding between her periods. It usually happens about a week before her “proper” period starts, and is quite light.  On questioning, she reports that her periods have also become increasingly painful over the past several years, with the pain starting a few days before her period and continuing until the bleeding has almost gone. She thought that this was just down to her age.  She had her first period aged 12. |
| **Absent findings** | No change in weight. Systemically well. |
| **Past medical and surgical history** | Nil significant |
| **Menstrual cycle length and regularity** | Average cycle length: 35  Cycle regularity (number of days difference between shortest and longest cycle in the past year): 5  Average period length: 5-7  Bleeding volume during period (low/medium/heavy, if heavy, is it ever enough to soak more than one tampon/pad every hour several hours in a row, clots): Medium  Any missed periods/periods of amenorrhea: None |
| **Menstrual pain or problems**  (e.g. any bleeding outside of period, any bloating or constipation and if it’s related to period timing, any association with bowel movements, urination, sex. For pain please include Site, Onset, Character, Radiation, Associations, Time course (e.g. cyclical nature or pattern), Exacerbating/ relieving factors) | Pain as above. She tends to get loose stools around the time of her period and reports crampy abdominal pain when this happens. She has no other gastrointestinal or urinary symptoms. |
| **Menstrual pain severity/frequency** (if applicable)  *Check the relevant box* | - No menstrual pain   Severity:  Not applicable  Mild  Moderate  Severe  Extremely severe  Frequency:  Never  Sometimes (once every 2-3 cycles)  Regularly (A few days every cycle)  Always (every cycle, almost all the time) |
| **Obstetric history**  (include time spent trying to conceive, if applicable) | Conceived within a year of trying at 22 and 24. Two normal vaginal deliveries. |
| **Gynae history**  (please include vaginal discharge characteristics if applicable including vaginal dryness) | As above only. No unusual discharge. |
| **Sexual history**  (include post-coital vaginal bleeding, dyspareunia - on initial penetration, deep penetration or both, vaginal dryness, changes in libido, or other sexual or contraception concerns) | Occasionally sexually active with casual partners and uses condoms. Has noticed some mild deep dyspareunia in recent years but thought this was due to her age. |
| **Family history** | Dad has type 2 diabetes and mum has hypertension. |
| **Any additional information**  (e.g. regularly bothered by gastrointestinal, urinary, mental/emotional issues, fatigue, sleep disturbances, changes in appetite or eating, skin changes - severe acne, hyperpigmentation, baldness, hirsutism (including location)) | None |
| **Impact of bleeding and pain on quality of life (if applicable)**  *Check the relevant box* | - No bleeding/pain   She is finding the unpredictable bleeding annoying and the pain with her periods is becoming increasingly difficult to manage.  Frequency:  No impact on quality of life  Sometimes (at least once per month, or once per 2-3 cycles)  Regularly (at least once per week, or a few days per cycle)  Always (almost every day, or every cycle almost all of the time) |

### Uterine Fibroids vignette cases: True positive, True negative, False negative, False positive

| **Condition** | Uterine Fibroids |
| --- | --- |
| **GP classification** | Strong match |
| **SC classification** | Strong match |
| **Classification agreement** | True positive |
|  |  |
| **Age** | 29 |
| **Sex** | Female |
| **BMI** | 28 |
| **Smoking status** | Non-smoker |
| **Alcohol intake (units per week)** | 18 |
| **Medication** | None |
| **LMP** | 10 days ago |
| **Gravidity** | 0 |
| **Parity** | 0 |
| **Chief complaints** | Bloating and abdominal swelling |
| **History of presenting illness**  (please indicate for all symptoms: duration and frequency. Specifically for pain, please include Site, Onset, Character, Radiation, Associations, Time course (e.g. cyclical nature or pattern), Exacerbating/ relieving factors, severity (e.g. mild, moderate, severe, extremely severe) | 7 month history.  Initially noticed mild bloating at times, especially in the evenings.  Now abdomen looks swollen most of the time - described as a ‘5 month pregnancy’.  Feeling of pressure on the bladder and needs to urinate frequently  Constant, mild discomfort in the lower abdomen. |
| **Absent findings** | Pregnancy test negative  Feels well in self  No weight loss  No change in bowel habit other than longstanding mild constipation at times (which responds well to dietary changes)  Energy levels good  No bleeding from bowel or bladder |
| **Past medical and surgical history** | Allergic to penicillin |
| **Menstrual cycle length and regularity** | Average cycle length: 27 days  Cycle regularity (number of days difference between shortest and longest cycle in the past year): 2 days  Average period length: 10 days  Bleeding volume during period (low/medium/heavy, if heavy, is it ever enough to soak more than one tampon/pad every hour several hours in a row, clots): Increasingly heavy. Needs to change pads every 15 minutes for a few hours on the first day each period. Doubles up on protection, and has frequent flooding.  Any missed periods/periods of amenorrhea: No |
| **Menstrual pain or problems**  (e.g. any bleeding outside of period, any bloating or constipation and if it’s related to period timing, any association with bowel movements, urination, sex. For pain please include Site, Onset, Character, Radiation, Associations, Time course (e.g. cyclical nature or pattern), Exacerbating/ relieving factors) | No unscheduled bleeding or bleeding after sex  Moderate period pain (longstanding) - 5/10 intensity, settles with paracetamol. |
| **Menstrual pain severity/frequency** (if applicable)  *Check the relevant box* | - No menstrual pain   Severity:  Not applicable  Mild  Moderate  Severe  Extremely severe  Frequency:  Never  Sometimes (once every 2-3 cycles)  Regularly (A few days every cycle)  Always (every cycle, almost all the time) |
| **Obstetric history**  (include time spent trying to conceive, if applicable) | None |
| **Gynae history**  (please include vaginal discharge characteristics if applicable including vaginal dryness) | None |
| **Sexual history**  (include post-coital vaginal bleeding, dyspareunia - on initial penetration, deep penetration or both, vaginal dryness, changes in libido, or other sexual or contraception concerns) | Some discomfort on deep penetration  No other changes  Not on any hormonal contraception |
| **Family history** | Mum, sister and two aunts all have/had fibroids |
| **Any additional information**  (e.g. regularly bothered by gastrointestinal, urinary, mental/emotional issues, fatigue, sleep disturbances, changes in appetite or eating, skin changes - severe acne, hyperpigmentation, baldness, hirsutism (including location)) | Nil of note |
| **Impact of bleeding and pain on quality of life (if applicable)**  *Check the relevant box* | - No bleeding/pain   Frequency:  No impact on quality of life  Sometimes (at least once per month, or once per 2-3 cycles)  Regularly (at least once per week, or a few days per cycle)  Always (almost every day, or every cycle almost all of the time) |

| **Condition** | Uterine Fibroids |
| --- | --- |
| **GP classification** | Weak match |
| **SC classification** | Weak match |
| **Classification agreement** | True negative |
|  |  |
| **Age** | 20 |
| **Sex** | Female |
| **BMI** | 28 |
| **Smoking status** | Non-smoker |
| **Alcohol intake (units per week)** | 12 |
| **Medication** | Combined oral contraceptive pill (rigevidon) |
| **LMP** | 1 week ago |
| **Gravidity** | 0 |
| **Parity** | 0 |
| **Chief complaints** | Discharge |
| **History of presenting illness**  (please indicate for all symptoms: duration and frequency. Specifically for pain, please include Site, Onset, Character, Radiation, Associations, Time course (e.g. cyclical nature or pattern), Exacerbating/ relieving factors, severity (e.g. mild, moderate, severe, extremely severe) | Thick, white, ‘cottage cheese’ vaginal discharge noted for the past week.  Accompanied by intense itch and irritated feeling.  Some discomfort on passing urine. |
| **Absent findings** | No unusual bleeding, no urinary frequency, no fever, no nausea or vomiting, no diarrhoea, no other symptoms or concerns |
| **Past medical and surgical history** | None - generally fit and well. |
| **Menstrual cycle length and regularity** | Average cycle length: 28  Cycle regularity (number of days difference between shortest and longest cycle in the past year): *No information*  Average period length: 1  Bleeding volume during period (low/medium/heavy, if heavy, is it ever enough to soak more than one tampon/pad every hour several hours in a row, clots): low  Any missed periods/periods of amenorrhea: No |
| **Menstrual pain or problems**  (e.g. any bleeding outside of period, any bloating or constipation and if it’s related to period timing, any association with bowel movements, urination, sex. For pain please include Site, Onset, Character, Radiation, Associations, Time course (e.g. cyclical nature or pattern), Exacerbating/ relieving factors) | None |
| **Menstrual pain severity/frequency** (if applicable)  *Check the relevant box* | No menstrual pain  Severity:   - Not applicable - Mild - Moderate - Severe - Extremely severe   Frequency:   - Never - Sometimes (once every 2-3 cycles) - Regularly (A few days every cycle) - Always (every cycle, almost all the time) |
| **Obstetric history**  (include time spent trying to conceive, if applicable) | None |
| **Gynae history**  (please include vaginal discharge characteristics if applicable including vaginal dryness) | None |
| **Sexual history**  (include post-coital vaginal bleeding, dyspareunia - on initial penetration, deep penetration or both, vaginal dryness, changes in libido, or other sexual or contraception concerns) | Currently uses combined contraceptive pill. Misses no more than one pill per month and follows missed pill rules when required. Has one long-term partner. |
| **Family history** | Nil of note |
| **Any additional information**  (e.g. regularly bothered by gastrointestinal, urinary, mental/emotional issues, fatigue, sleep disturbances, changes in appetite or eating, skin changes - severe acne, hyperpigmentation, baldness, hirsutism (including location)) | Has previously had thrush, treated successfully with over-the-counter Canesten. |
| **Impact of bleeding and pain on quality of life (if applicable)**  *Check the relevant box* | No bleeding/pain  Frequency:   - No impact on quality of life - Sometimes (at least once per month, or once per 2-3 cycles) - Regularly (at least once per week, or a few days per cycle) - Always (almost every day, or every cycle almost all of the time) |

| **Condition** | Uterine Fibroids |
| --- | --- |
| **GP classification** | Strong match |
| **SC classification** | Weak match |
| **Classification agreement** | False negative |
|  |  |
| **Age** | 44 |
| **Sex** | Female |
| **BMI** | 32 |
| **Smoking status** | Non smoker |
| **Alcohol intake (units per week)** | 0 |
| **Medication** | Metformin |
| **LMP** | 2 weeks ago |
| **Gravidity** | 0 |
| **Parity** | 0 |
| **Chief complaints** | Constipation and pain opening bowels during periods  Heavy, painful periods |
| **History of presenting illness**  (please indicate for all symptoms: duration and frequency. Specifically for pain, please include Site, Onset, Character, Radiation, Associations, Time course (e.g. cyclical nature or pattern), Exacerbating/ relieving factors, severity (e.g. mild, moderate, severe, extremely severe) | Had heavy and painful periods since menarche age 12 years old.  Bloating and constipation getting worse the last 3 years. Opens bowels every 3-4 days. Painful to open bowels. Worse during the period. Not relieved by over the counter laxatives. |
| **Absent findings** | None |
| **Past medical and surgical history** | Type 2 diabetes mellitus |
| **Menstrual cycle length and regularity** | Average cycle length: 26  Cycle regularity (number of days difference between shortest and longest cycle in the past year): 1  Average period length:7  Bleeding volume during period (low/medium/heavy, if heavy, is it ever enough to soak more than one tampon/pad every hour several hours in a row, clots):Heavy  Any missed periods/periods of amenorrhea:No |
| **Menstrual pain or problems**  (e.g. any bleeding outside of period, any bloating or constipation and if it’s related to period timing, any association with bowel movements, urination, sex. For pain please include Site, Onset, Character, Radiation, Associations, Time course (e.g. cyclical nature or pattern), Exacerbating/ relieving factors) | Moderate period pain, abdominal and radiates to the back passage (anus). Bloating relieved by opening bowels. Exacerbated during period.  Normal urination. Normal sexual intercourse. |
| **Menstrual pain severity/frequency** (if applicable)  *Check the relevant box* | - No menstrual pain   Severity:  Not applicable  Mild  Moderate  Severe  Extremely severe  Frequency:  Never  Sometimes (once every 2-3 cycles)  Regularly (A few days every cycle)  Always (every cycle, almost all the time) |
| **Obstetric history**  (include time spent trying to conceive, if applicable) | None |
| **Gynae history**  (please include vaginal discharge characteristics if applicable including vaginal dryness) | None |
| **Sexual history**  (include post-coital vaginal bleeding, dyspareunia - on initial penetration, deep penetration or both, vaginal dryness, changes in libido, or other sexual or contraception concerns) | Not sexually active |
| **Family history** | Mother has fibroids |
| **Any additional information**  (e.g. regularly bothered by gastrointestinal, urinary, mental/emotional issues, fatigue, sleep disturbances, changes in appetite or eating, skin changes - severe acne, hyperpigmentation, baldness, hirsutism (including location)) | Generally constipated. Associated bloating, worse during period. Abdomen can swell in size during period. No nausea/ vomiting. Normal appetite. Fatigue during period. Normal mood. No sleep disturbance. No skin changes. |
| **Impact of bleeding and pain on quality of life (if applicable)**  *Check the relevant box* | - No bleeding/pain   Frequency:  No impact on quality of life  Sometimes (at least once per month, or once per 2-3 cycles)  Regularly (at least once per week, or a few days per cycle)  Always (almost every day, or every cycle almost all of the time) |

| **Condition** | Uterine Fibroids |
| --- | --- |
| **GP classification** | Weak match |
| **SC classification** | Strong match |
| **Classification agreement** | False positive |
|  |  |
| **Age** | 36 |
| **Sex** | Female |
| **BMI** | 40 |
| **Smoking status** | Non-smoker |
| **Alcohol intake (units per week)** | 2 |
| **Medication** | Lymecycline 408mg OD |
| **LMP** | 3 months ago |
| **Gravidity** | 0 |
| **Parity** | 0 |
| **Chief complaints** | Heavy periods |
| **History of presenting illness**  (please indicate for all symptoms: duration and frequency. Specifically for pain, please include Site, Onset, Character, Radiation, Associations, Time course (e.g. cyclical nature or pattern), Exacerbating/ relieving factors, severity (e.g. mild, moderate, severe, extremely severe) | Periods have been heavy and unpredictable for the past few years.  Bleeds onto clothes and bedlinen regularly despite doubling up on protection.  Passes clots for the first few days of each cycle.  Misses at least 2 days of work per cycle due to heavy bleeding.  Finds symptoms distressing due to level of disruption.  Occasional bloating, not related to cycle. |
| **Absent findings** | No abdominal pain  No weight change  No bleeding between periods or after sex  No changes to bowels or bladder  Feels well in self otherwise. |
| **Past medical and surgical history** | Acne - on lymecycline |
| **Menstrual cycle length and regularity** | Average cycle length: 3 months  Cycle regularity (number of days difference between shortest and longest cycle in the past year): 100 days  Average period length: 10 days  Bleeding volume during period (low/medium/heavy, if heavy, is it ever enough to soak more than one tampon/pad every hour several hours in a row, clots): Heavy, as above  Any missed periods/periods of amenorrhea: Up to 6 months amenorrhoea |
| **Menstrual pain or problems**  (e.g. any bleeding outside of period, any bloating or constipation and if it’s related to period timing, any association with bowel movements, urination, sex. For pain please include Site, Onset, Character, Radiation, Associations, Time course (e.g. cyclical nature or pattern), Exacerbating/ relieving factors) | No other concerns.  No period pain.  No problems with bowels or bladder. |
| **Menstrual pain severity/frequency** (if applicable)  *Check the relevant box* | No menstrual pain  Severity:   - Not applicable - Mild - Moderate - Severe - Extremely severe   Frequency:  Never  Sometimes (once every 2-3 cycles)  Regularly (A few days every cycle)  Always (every cycle, almost all the time) |
| **Obstetric history**  (include time spent trying to conceive, if applicable) | None |
| **Gynae history**  (please include vaginal discharge characteristics if applicable including vaginal dryness) | Nil of note. |
| **Sexual history**  (include post-coital vaginal bleeding, dyspareunia - on initial penetration, deep penetration or both, vaginal dryness, changes in libido, or other sexual or contraception concerns) | Nil of note. |
| **Family history** | Father has ischaemic heart disease (diagnosed aged 69) |
| **Any additional information**  (e.g. regularly bothered by gastrointestinal, urinary, mental/emotional issues, fatigue, sleep disturbances, changes in appetite or eating, skin changes - severe acne, hyperpigmentation, baldness, hirsutism (including location)) | Finding it increasingly difficult to lose weight.  Some acne but managed reasonably well with antibiotics from GP.  Some bloating but no apparent pattern. |
| **Impact of bleeding and pain on quality of life (if applicable)**  *Check the relevant box* | No bleeding/pain  Frequency:  No impact on quality of life  Sometimes (at least once per month, or once per 2-3 cycles)  Regularly (at least once per week, or a few days per cycle)  Always (almost every day, or every cycle almost all of the time) |

### Polycystic ovary syndrome (PCOS) vignette cases: True positive, True negative, False positive

| **Condition** | Polycystic ovary syndrome (PCOS) |
| --- | --- |
| **GP classification** | Strong match |
| **SC classification** | Strong match |
| **Classification agreement** | True positive |
|  |  |
| **Age** | 32 |
| **Sex** | Female |
| **BMI** | 27 |
| **Smoking status** | Non smoker |
| **Alcohol intake (units per week)** | 0 |
| **Medication** | 0 |
| **LMP** | 6 weeks ago |
| **Gravidity** | 0 |
| **Parity** | 0 |
| **Chief complaints** | Difficulty trying to conceive  Irregular periods |
| **History of presenting illness**  (please indicate for all symptoms: duration and frequency. Specifically for pain, please include Site, Onset, Character, Radiation, Associations, Time course (e.g. cyclical nature or pattern), Exacerbating/ relieving factors, severity (e.g. mild, moderate, severe, extremely severe) | Trying to conceive for 2 years.  Regular sexual partner and having sexual intercourse every 2-3 days without contraception.  In past used condoms but never used other types of contraception.  Also has irregular and light periods. Every 2-3 months for the last 6 years |
| **Absent findings** | No period pain |
| **Past medical and surgical history** | None |
| **Menstrual cycle length and regularity** | Average cycle length:41 days  Cycle regularity (number of days difference between shortest and longest cycle in the past year): 14 days  Average period length:3 days  Bleeding volume during period (low/medium/heavy, if heavy, is it ever enough to soak more than one tampon/pad every hour several hours in a row, clots): light  Any missed periods/periods of amenorrhea: *No information* |
| **Menstrual pain or problems**  (e.g. any bleeding outside of period, any bloating or constipation and if it’s related to period timing, any association with bowel movements, urination, sex. For pain please include Site, Onset, Character, Radiation, Associations, Time course (e.g. cyclical nature or pattern), Exacerbating/ relieving factors) | No intermenstrual bleeding.  No bloating or constipation.  Normal urination.  Regular sexual intercourse, no pain. |
| **Menstrual pain severity/frequency** (if applicable)  *Check the relevant box* | No menstrual pain  Severity:   - Not applicable - Mild - Moderate - Severe - Extremely severe   Frequency:   - Never - Sometimes (once every 2-3 cycles) - Regularly (A few days every cycle) - Always (every cycle, almost all the time) |
| **Obstetric history**  (include time spent trying to conceive, if applicable) | Never been pregnant. Trying to conceive for 2 years. |
| **Gynae history**  (please include vaginal discharge characteristics if applicable including vaginal dryness) | None |
| **Sexual history**  (include post-coital vaginal bleeding, dyspareunia - on initial penetration, deep penetration or both, vaginal dryness, changes in libido, or other sexual or contraception concerns) | Long term partner.  No dyspareunia  No post coital bleeding.  Normal libido.  No dryness. |
| **Family history** | None |
| **Any additional information**  (e.g. regularly bothered by gastrointestinal, urinary, mental/emotional issues, fatigue, sleep disturbances, changes in appetite or eating, skin changes - severe acne, hyperpigmentation, baldness, hirsutism (including location)) | Severe acne on face and back  Hirsutism on chest and chin |
| **Impact of bleeding and pain on quality of life (if applicable)**  *Check the relevant box* | No bleeding/pain  Frequency:   - No impact on quality of life - Sometimes (at least once per month, or once per 2-3 cycles) - Regularly (at least once per week, or a few days per cycle) - Always (almost every day, or every cycle almost all of the time) |

| **Condition** | Polycystic ovary syndrome (PCOS) |
| --- | --- |
| **GP classification** | Weak match |
| **SC classification** | Weak match |
| **Classification agreement** | True negative |
|  |  |
| **Age** | 24 |
| **Sex** | Female |
| **BMI** | 26.5 |
| **Smoking status** | Smoker |
| **Alcohol intake (units per week)** | 0 |
| **Medication** | Nil |
| **LMP** | 1 week ago |
| **Gravidity** | 0 |
| **Parity** | 0 |
| **Chief complaints** | Bloating and abdominal discomfort |
| **History of presenting illness**  (please indicate for all symptoms: duration and frequency. Specifically for pain, please include Site, Onset, Character, Radiation, Associations, Time course (e.g. cyclical nature or pattern), Exacerbating/ relieving factors, severity (e.g. mild, moderate, severe, extremely severe) | 6 month history of abdominal bloating and discomfort associated with mealtimes and relieved after opening bowels. Sometimes associated with loose stool. |
| **Absent findings** | No blood/weight loss/fatigue. |
| **Past medical and surgical history** | Nil |
| **Menstrual cycle length and regularity** | Average cycle length: 32 days  Cycle regularity (number of days difference between shortest and longest cycle in the past year): 5 days  Average period length: 2-3 days  Bleeding volume during period (low/medium/heavy, if heavy, is it ever enough to soak more than one tampon/pad every hour several hours in a row, clots): medium  Any missed periods/periods of amenorrhea: No |
| **Menstrual pain or problems**  (e.g. any bleeding outside of period, any bloating or constipation and if it’s related to period timing, any association with bowel movements, urination, sex. For pain please include Site, Onset, Character, Radiation, Associations, Time course (e.g. cyclical nature or pattern), Exacerbating/ relieving factors) | No other menstrual complaints. |
| **Menstrual pain severity/frequency** (if applicable)  *Check the relevant box* | No menstrual pain  Severity:   - Not applicable - Mild - Moderate - Severe - Extremely severe   Frequency:  Never  Sometimes (once every 2-3 cycles)  Regularly (A few days every cycle)  Always (every cycle, almost all the time) |
| **Obstetric history**  (include time spent trying to conceive, if applicable) | None. |
| **Gynae history**  (please include vaginal discharge characteristics if applicable including vaginal dryness) | None. |
| **Sexual history**  (include post-coital vaginal bleeding, dyspareunia - on initial penetration, deep penetration or both, vaginal dryness, changes in libido, or other sexual or contraception concerns) | No sexual or contraception concerns. |
| **Family history** | None. |
| **Any additional information**  (e.g. regularly bothered by gastrointestinal, urinary, mental/emotional issues, fatigue, sleep disturbances, changes in appetite or eating, skin changes - severe acne, hyperpigmentation, baldness, hirsutism (including location)) | None. |
| **Impact of bleeding and pain on quality of life (if applicable)**  *Check the relevant box* | No bleeding/pain  Frequency:  No impact on quality of life  Sometimes (at least once per month, or once per 2-3 cycles)  Regularly (at least once per week, or a few days per cycle)  Always (almost every day, or every cycle almost all of the time) |

| **Condition** | Polycystic ovary syndrome (PCOS) |
| --- | --- |
| **GP classification** | Weak match |
| **SC classification** | Strong match |
| **Classification agreement** | False positive |
|  |  |
| **Age** | 34 |
| **Sex** | Female |
| **BMI** | 26 |
| **Smoking status** | None smoker |
| **Alcohol intake (units per week)** | 10 |
| **Medication** | None |
| **LMP** | 5 weeks ago |
| **Gravidity** | 0 |
| **Parity** | 0 |
| **Chief complaints** | Irregular periods |
| **History of presenting illness**  (please indicate for all symptoms: duration and frequency. Specifically for pain, please include Site, Onset, Character, Radiation, Associations, Time course (e.g. cyclical nature or pattern), Exacerbating/ relieving factors, severity (e.g. mild, moderate, severe, extremely severe) | Irregular and heavier periods over the last 12 months.  Periods every 5-6 weeks compared to previously every 4 weeks. |
| **Absent findings** | No period pain. No acne, no hirsutism, no bloating, no pain on opening bowels or urinating. |
| **Past medical and surgical history** | None |
| **Menstrual cycle length and regularity** | Average cycle length: 5 weeks  Cycle regularity (number of days difference between shortest and longest cycle in the past year): 10  Average period length:7  Bleeding volume during period (low/medium/heavy, if heavy, is it ever enough to soak more than one tampon/pad every hour several hours in a row, clots): heavy  Any missed periods/periods of amenorrhea: no |
| **Menstrual pain or problems**  (e.g. any bleeding outside of period, any bloating or constipation and if it’s related to period timing, any association with bowel movements, urination, sex. For pain please include Site, Onset, Character, Radiation, Associations, Time course (e.g. cyclical nature or pattern), Exacerbating/ relieving factors) | No menstrual pain  Normal urination  Constipated - opens bowels every 2 days with hard stools.  Normal sexual intercourse |
| **Menstrual pain severity/frequency** (if applicable)  *Check the relevant box* | No menstrual pain  Severity:   - Not applicable - Mild - Moderate - Severe - Extremely severe   Frequency:   - Never - Sometimes (once every 2-3 cycles) - Regularly (A few days every cycle) - Always (every cycle, almost all the time) |
| **Obstetric history**  (include time spent trying to conceive, if applicable) | None |
| **Gynae history**  (please include vaginal discharge characteristics if applicable including vaginal dryness) | None |
| **Sexual history**  (include post-coital vaginal bleeding, dyspareunia - on initial penetration, deep penetration or both, vaginal dryness, changes in libido, or other sexual or contraception concerns) | None |
| **Family history** | Mother has hypothyroidism |
| **Any additional information**  (e.g. regularly bothered by gastrointestinal, urinary, mental/emotional issues, fatigue, sleep disturbances, changes in appetite or eating, skin changes - severe acne, hyperpigmentation, baldness, hirsutism (including location)) | Hair and skin dry  Put on weight over last 6 months.  Feels cold all the time.  Fatigue and low mood |
| **Impact of bleeding and pain on quality of life (if applicable)**  *Check the relevant box* | No bleeding/pain  Frequency:   - No impact on quality of life - Sometimes (at least once per month, or once per 2-3 cycles) - Regularly (at least once per week, or a few days per cycle) - Always (almost every day, or every cycle almost all of the time) |
